# Rudra Interrupts Receptor Signaling Complexes to Negatively Regulate the IMD Pathway

**DOI:** 10.1371/journal.ppat.1000120

**Published:** 2008-08-08

**Authors:** Kamna Aggarwal, Florentina Rus, Christie Vriesema-Magnuson, Deniz Ertürk-Hasdemir, Nicholas Paquette, Neal Silverman

**Affiliations:** Divison of Infectious Diseases, Department of Medicine, University of Massachusetts Medical School, Worcester, Massachusetts, United States of America; Stanford University, United States of America

## Abstract

Insects rely primarily on innate immune responses to fight pathogens. In *Drosophila*, antimicrobial peptides are key contributors to host defense. Antimicrobial peptide gene expression is regulated by the IMD and Toll pathways. Bacterial peptidoglycans trigger these pathways, through recognition by peptidoglycan recognition proteins (PGRPs). DAP-type peptidoglycan triggers the IMD pathway via PGRP-LC and PGRP-LE, while lysine-type peptidoglycan is an agonist for the Toll pathway through PGRP-SA and PGRP-SD. Recent work has shown that the intensity and duration of the immune responses initiating with these receptors is tightly regulated at multiple levels, by a series of negative regulators. Through two-hybrid screening with PGRP-LC, we identified Rudra, a new regulator of the IMD pathway, and demonstrate that it is a critical feedback inhibitor of peptidoglycan receptor signaling. Following stimulation of the IMD pathway, *rudra* expression was rapidly induced. In cells, RNAi targeting of *rudra* caused a marked up-regulation of antimicrobial peptide gene expression. *rudra* mutant flies also hyper-activated antimicrobial peptide genes and were more resistant to infection with the insect pathogen *Erwinia carotovora carotovora*. Molecularly, Rudra was found to bind and interfere with both PGRP-LC and PGRP-LE, disrupting their signaling complex. These results show that Rudra is a critical component in a negative feedback loop, whereby immune-induced gene expression rapidly produces a potent inhibitor that binds and inhibits pattern recognition receptors.

## Author Summary

The innate immune system controls the immediate response to infection. Innate immunity relies on germline encoded receptors, receptors that are present at birth, to recognize germs and trigger a protective response. Invertebrates (i.e., insects) rely on innate immunity to survive in microbial-rich environments, such as rotting fruit. However, uncontrolled innate immune responses are dangerous, leading to severe pathologies like sepsis, inflammatory bowel diseases, and lupus. Therefore, the intensity and duration of the innate immune response is kept in-check by multiple regulatory mechanisms. Here, we have identified a new feedback regulator of the *Drosophila* (the fruit fly) immune response, which we call Rudra. Using various approaches, we show that in the absence of Rudra the innate immune system is hyper-activated. This elevated immune response leads to better protection against bacterial infection. On the other hand, when present in excess, Rudra prevents the activation of the immune response. Furthermore, we show that Rudra turns off the immune response by binding to the receptors that are responsible for detecting bacteria, thereby preventing downstream responses.

## Introduction

Insects rely primarily on innate immune responses to fight pathogens. The *Drosophila* immune response has proven to be an experimentally powerful and conserved model system for the study of innate immunity [1],[2],[3],[4]. In particular, the insect immune response relies on evolutionary conserved NF-κB signaling cascades for the control of inducible antimicrobial peptide (AMP) gene transcription. This antimicrobial peptide response is critical for protection against many microbial pathogens [5],[6].

In *Drosophila*, two signaling pathways regulate the production of these antimicrobial peptides - the IMD and Toll pathways [7]. The Toll pathway responds to many Gram-positive bacterial and fungal infections [8], while the IMD pathway is potently activated by DAP-type peptidoglycan (PGN) from Gram-negative bacteria and certain Gram-positive bacteria [9],[10]. Two receptors, PGRP-LC and PGRP-LE, are able to recognize DAP-type PGN at the cell surface or in the cytosol, respectively, and trigger the IMD pathway [11],[12],[13],[14],[15],[16].

Upon binding DAP-type PGN, both PGRP-LC and PGRP-LE multimerize and signal via a common motif in their N-terminal domains, known as the RHIM-like domain [15],[17],[18]. The RHIM-like domain is critical for signaling by either receptor, but the mechanism(s) involved remain unclear [15]. Genetic experiments suggest that the *imd* protein functions immediately downstream of PGRP-LC and upstream of all other known components of the pathway [19]. IMD associates with both PGRP-LC and -LE, although the PGRP-LC RHIM-like motif is not required for this interaction [15]. Nonetheless, the complexes formed on these receptors are likely to be critical to trigger further signal transduction.

Recent work has shown that the intensity and duration of the immune response is tightly regulated in *Drosophila*. As in mammals, over-exuberant immune responses can be detrimental, and the proper down modulation of immunity is critical for health and fecundity [20],[21],[22]. In order to keep the immune response properly modulated, the Toll and IMD pathways are controlled at multiple levels by a series of negative regulators. For example, the amidases PGRP-LB and PGRP-SC reduce the immunostimulatory activity of PGN by digesting it [23],[24]. Intracellularly, the IMD signaling pathway is further down–regulated by Dnr1, POSH, Caspar and the E3-ligase complex containing SkpA, dCullin and Slimb [25],[26],[27],[28]. Additionally, the JNK and Relish branches of the IMD pathway are thought to mutually inhibit each other [29],[30],[31].

In this study, we identify and characterize a negative feedback regulator of the IMD pathway, dubbed *rudra*. Expression of *rudra* was rapidly induced following immune challenge. Moreover, in flies and cells, *rudra* is critical for controlling immune-induced gene expression. Following infection, *rudra* mutant flies hyper-activated antimicrobial peptide gene expression resulting in increased resistance to microbial infection. Using various biochemical and genetic techniques, Rudra was found to interact with the receptors PGRP-LC and PGRP-LE and disrupt the signaling complex assembled on these receptors. Due to its ability to destroy this receptor signaling complex and inhibit immune responses, *rudra* was named for Shiva, the Indian god of destruction, who in his Rudra phase of mind causes inhibition and destruction of all life on earth.

## Results

### Isolation of Rudra

In order to identify potential partners and regulators of the IMD pathway receptors, a yeast two-hybrid screen was performed with the cytoplasmic domain of PGRP-LC as bait [32],[33]. 25 strongly interacting clones were further analyzed with a set of baits that carried mutations in the RHIM-like domain of PGRP-LC (or irrelevant control baits). One clone interacted strongly with the wild-type cytoplasmic domain of PGRP-LC but weakly with the RHIM-like mutant baits (Table 1). This clone encoded amino acids 30–197 of CG15678, and will be referred to as *rudra* from hereafter.

**Table 1:** Rudra interacts with cytoplasmic domain of PGRP-LC by yeast two-hybrid.

Baits	LC WT	LC Δ172-212	LC Δ213-242	LC F218A	DmIKK	Empty vector
Prey: Rudra aa 30–197	++++	++	++	++	−	−

The cytoplasmic domain of PGRP-LC was used as bait and Rudra was used as the prey in yeast two-hybrid assays. Rudra interacted well with the full cytoplasmic domain of PGRP-LC and the yeast cells grew robustly on Ade selection plates. However, Rudra interacted weakly with several deletion and point mutants that alter the RHIM-like domain of PGRP-LCx. ++++, robust growth; ++, slow growth, − no growth.

**Figure 1:**
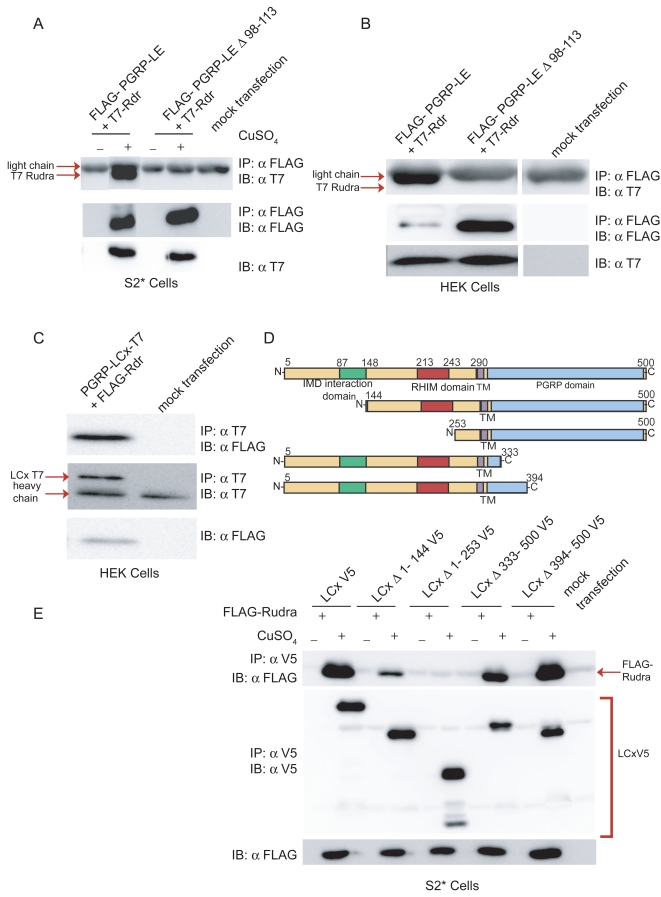
Rudra interacts with the receptors PGRP-LE and PGRP-LC. (A, B) Immunoprecipitation (IP) and immunoblot (IB) analysis of lysates from S2^*^ or HEK cells transiently transfected with expression plasmids for FLAG-tagged *PGRP-LE* and/or T7-tagged *rudra*. In the *Drosophila* S2* cells, the copper inducible metallothionein promoter was used for expression and cells were treated with CuSO_4_ or left untreated, as indicated. (C) Similar co-immunoprecipitation experiments from lysates of HEK cells transiently co-transfected with T7-tagged *PGRP-LCx* and FLAG-tagged *rudra* expression plasmids. (D) Schematic representation of the PGRP-LCx deletions mutants used in (E). (E) IP-IB analysis of lysates from S2^*^ cells transiently transfected with metallothionein promoter expression plasmids encoding wild-type and deletion mutants of V5-tagged *PGRP-LCx* and FLAG-tagged *rudra*, with or without CuSO_4_treatment, as indicated. Data are representative of at least three independent assays.

To confirm the yeast two-hybrid results, co-immunoprecipitation experiments were performed. Using epitope tagged constructs and transient transfection in *Drosophila* S2* cells, both PGRP-LE and PGRP-LC were found to associate with Rudra (Figure 1A, E). In a heterologous system (HEK cells), similar robust associations were observed between Rudra and PGRP-LE or −LC (Figure 1B, C). The interaction between Rudra and PGRP-LE was also readily detectable, by co-immunoprecipitation, when these proteins were produced in a rabbit reticulocyte *in vitro* translation system (Figure S1). These data demonstrate that Rudra interacts directly with the receptors PGRP-LC and PGRP-LE.

In order to determine which domain(s) of the receptors interact with Rudra, co-immunoprecipitation assays were performed with various mutant versions of PGRP-LC or PGRP-LE. Consistent with the yeast two-hybrid data, which indicated involvement of the RHIM-like domain for interaction, a mutant form of PGRP-LE lacking the RHIM motif (Δ98-113) showed little interaction with Rudra (Figure 1A, B). Using a set of large deletions (Figure 1D), the N-terminal cytoplasmic domain of PGRP-LC was found to be essential for association with Rudra. Removal of the first 144 amino acids decreased Rudra interaction, while removal of nearly the entire cytoplasmic (Δ1-253) domain abolished interaction. The PGRP-LC extracellular domain was not involved in the interaction (Figure 1E). We then attempted to map the PGRP-LC interaction more finely with a set of mutants that span the entire cytoplasmic domain with sequential 50 amino acid deletions. However, Rudra co-immunoprecipitated with all of these deletion mutants, suggesting some redundancy in the interaction mechanism (Figure S2). The yeast two-hybrid data suggest that some of the interacting activity involves the PGRP-LC RHIM domain, while the larger deletions suggest another interaction motif likely lies in the first 144 amino acids (Figure 1D, E). Overall, we conclude that Rudra directly interacts with the signaling domains of PGRP-LC and PGRP-LE. The interaction with PGRP-LE is largely mediated by the RHIM motif while the interaction with PGRP-LC appears to involve multiple, partly redundant, mechanisms.

### Induction of *rudra* expression

**Figure 2:**
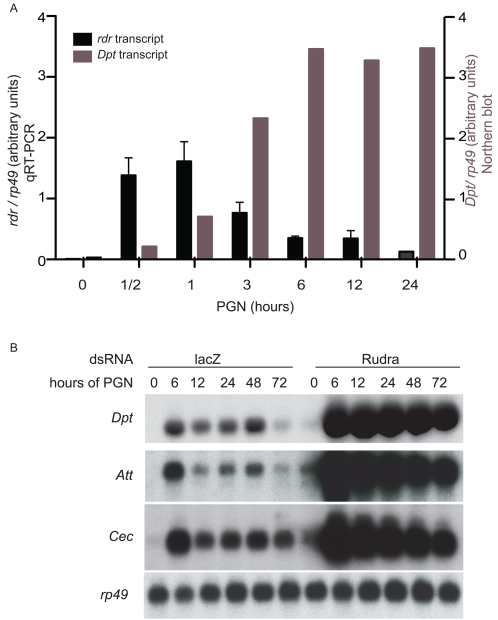
*rudra*, a negative feedback regulator of IMD signaling in cells. (A) Real-time RT-PCR analysis of *rudra* transcript from S2* cells which were stimulated with PGN for various times. *Diptericin* expression was quantified, by Northern blot, from these same cells. (B) Northern blot of *Diptericn*, *Attacin*, *Cecropin* and *rp49* expression in S2* cells treated with lacZ dsRNA or Rudra dsRNA, and then stimulated with PGN for various times. Data are representative of at least three independent assays. Error bars in (A) represent standard deviation on 3 technical repeats.

Previous microarray studies have suggested that *rudra* is a target of the IMD signaling pathway [29],[34],[35]. In order to confirm and extend these findings, the expression of *rudra* was analyzed at various times after immune stimulation of S2* cells, by qRT-PCR. *rudra* transcript was rapidly induced, peaking in 30–60 minutes and returning to near baseline levels within 24 hours (Figure 2A). The kinetics of *rudra* expression were markedly faster and more transient than the expression of AMP genes. For example, *Diptericin* mRNA levels, as measured by Northern blotting, did not peak until 6 hours after PGN stimulation, and then remained elevated for at least 24 hours (Figure 2A). Even though the expression profiles of *rudra* and AMP genes are distinct, they both require the NF-κB factor Relish [35],[36].

### Rudra is a negative regulator of IMD signaling

Next, RNAi was used to characterize the function of *rudra* in the IMD pathway. S2* cells were transfected with dsRNA for *rudra*, and then stimulated with PGN for various times. As monitored by Northern blotting, antimicrobial peptide genes *Diptericin (Dpt)*, *Attacin (Att)* and *Cecropin (Cec)* were induced to markedly higher levels in cells treated with *rudra* RNAi, compared to cells transfected with a control *lacZ* dsRNA (Figure 2B). These data suggest that *rudra* is a negative regulator of IMD signaling.

**Figure 3:**
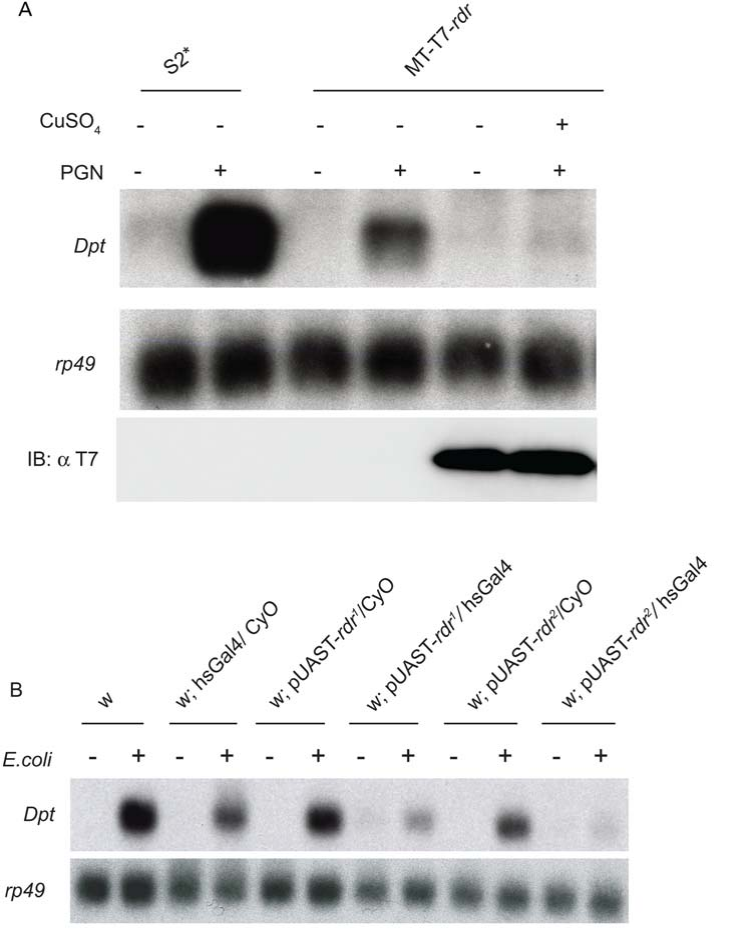
Over-expression of *rudra* blocks IMD signaling in both cells and flies. (A) Northern blot of *Dpt* and *rp49* expression in S2^*^ cells stably transfected with a metallothionein promoter–driven transgene expressing *rudra*. Cells were treated with CuSO_4_ for 1.5 hours and then stimulated with PGN for 5 hours, as indicated. (B) Northern blot of *Diptericin* and *rp49* expression in adult flies carrying UAS promoter–driven transgenes expressing *rudra* (two independent transgenic lines). Flies were heat shocked for 1.5 hours and then RNA was isolated 8 hours after septic infection with *E.coli*. Data are representative of at least three independent assays.

To further test if *rudra* is a negative regulator of the IMD pathway, stable cell lines expressing *rudra* from a copper-inducible promoter were selected. These cell lines were treated with copper for 1.5 hours, to induce *rudra* expression, and then stimulated with PGN for 5 hours, to stimulate the IMD pathway. *rudra* over-expression potently inhibited the induction of *Dpt* (Figure 3A). Also, to test if *rudra* negatively regulates the Toll pathway, stable cell lines expressing *rudra* from the actin promoter were selected. These cell lines were treated with SPZ-C106 for 18 hours to stimulate the Toll pathway. *rudra* over-expression did not robustly inhibit the induction of *Drosomycin*, as compared to its ability to inhibit PGN-induced *Diptericin* expression (Figure S3). These data demonstrate that *rudra* is potent inhibitor of the IMD pathway but has little effect on Toll signaling.

Using the UAS system and a heat shock Gal4 ‘driver’, transgenic flies that ectopically express *rudra* were also characterized. *rudra e*xpression was induced with a 1.5 hour heat shock and then flies were challenged with *E.coli*. In two independent UAS-*rudra* lines, IMD signaling was strongly inhibited by *rudra* expression, as monitored by Northern blotting for *Dpt* induction (Figure 3A). These results are consistent with the data from cultured cells, and argue that *rudra* is a potent negative regulator of the IMD pathway *in vivo*.

**Figure 4:**
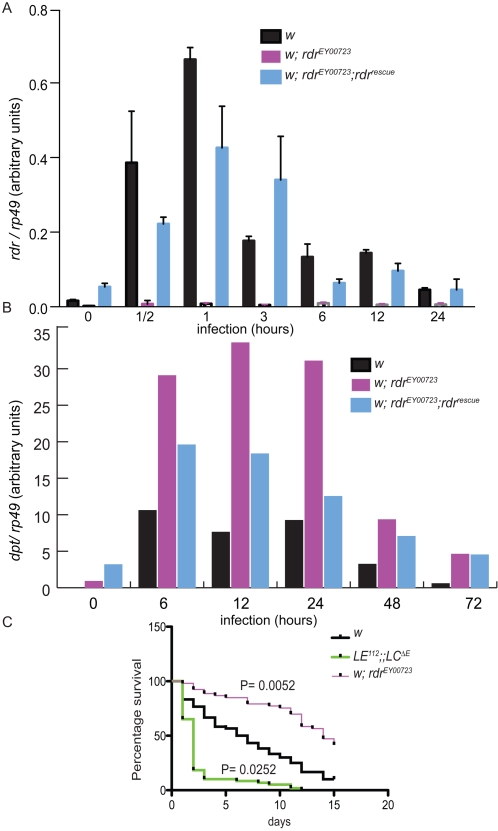
Characterization of *rudra* mutant flies. (A) Real-time RT-PCR analysis of *rudra* transcript from *w^1118^*, *rudra^EY00723^*, and *rdr^rescue^* flies that were infected with *E.coli* for various times. (B) Quantified Northern blotting data of *Diptericin* and *rp49* expression in *w^1118^*, *rudra^EY00723^* and *rudra^rescue^* flies following infection with *E.coli*. (C) Survival assays were performed following infection of *w^1118^*, *rudra^EY00723^* and *LE^112^;LC^ΔE^* flies with *E. carotovora carotovora*. Infected animals were incubated at 29°C and the number of surviving flies were counted every 24 hours. Survival data is presented in Kaplan-Meier plots and significance was analyzed by log-rank test. (A) and (B) are representative of at least 3 independent experiments, while (C) is representative of 2 independent trials, with 60 or 100 animals.

In order to phenotypically characterize the loss of *rudra*, a strain carrying a P-element at position 123 in the 5′ UTR of *rudra* (EY00723) was analyzed [37],[38],[39]. First, the level of *rudra* transcript in this strain was compared to an isogenic *white* strain, by qRT-PCR (Figure 4A). [To isogenize mutant and wild-type strains, EY00723 was backcrossed with the *white* strain for six generations prior to these analyses]. Similar to the cell culture data, *rudra* transcription was rapidly induced following infection in wild-type flies. Again, the induction of *rudra* expression occurs more rapidly, and is resolved more quickly, than does AMP gene expression (compare Figure 4A to 4B). The transposon insertion in the 5′ UTR markedly inhibited *rudra* expression, with nearly undetectable levels at all time points, demonstrating that this allele of *rudra* is a strong hypomorph. Also, a transgenic rescue strain was constructed, using a 4.5 Kbp genomic fragment (*rudra^rescue^*). This genomic rescue construct partially restored immune-inducible expression of *rudra*, but it did not completely return to wild-type levels (Figure 4A).

Next, the immune response of wild-type, *rudra^EY00723^*, and the *rudra^rescue^* strains were compared. *Diptericin* expression, as monitored by Northern blotting at various times following septic *E. coli* infection, was elevated at all time points in *rudra^EY00723^* compared to the isogenic wild-type strain (Figure 4B). The *rudra^rescue^* transgenic line restored *Diptericin* to levels between that observed in the wild-type and *rudra* mutant flies, consistent with partially restored levels of *rudra* expression observed in this line. *rudra* heterozygotes also displayed elevated AMP gene expression (data not shown). These results, together with the data from ectopic expression, demonstrate that *rudra* is a potent negative regulator of the IMD pathway in flies, as well as in cultured cell lines.

We then asked what consequence these elevated AMP levels might have during an infection. To this end, wild-type and *rudra^EY00723^* flies were infected with the Gram-negative pathogen *Erwinia carotovora carotovora (Ecc)*. As reported previously, *Ecc* is a mildly pathogenic infection in wild-type animals, such that most flies succumb over the course ∼10 days (Figure 4C) [27],[40]. As expected, PGRP-*LE; PGRP-LC double* mutant flies, which lack both receptors involved in detecting DAP-type PGN, were rapidly killed by this infection (P = 0.0252, compared to wild-type animals). On the other hand, *rudra* mutants showed significantly improved survival compared to wild-type flies (P = 0.0052). These results show that loss of *rudra*, and the ensuing increase in AMP levels, enhances resistance to this Gram-negative pathogen.

### Rudra inhibits signaling at the receptor

**Figure 5:**
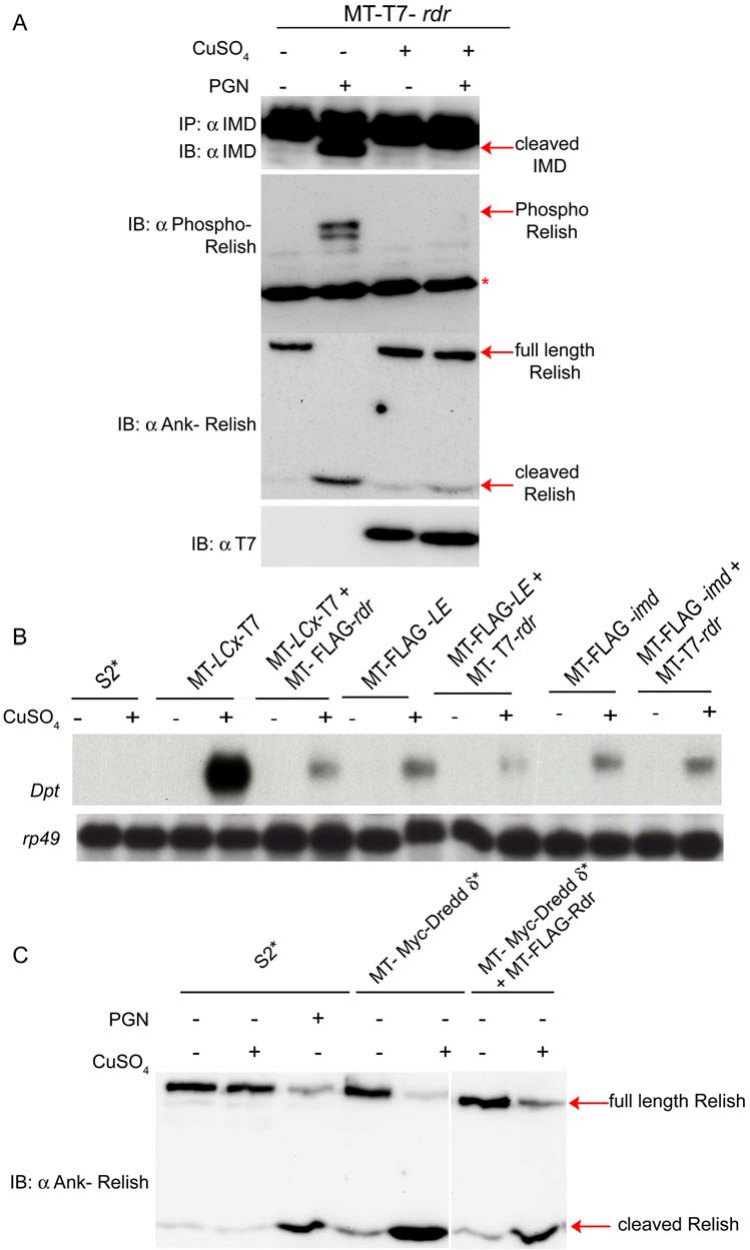
Rudra functions upstream of IMD, Dredd and Relish. (A) Analysis of lysates from S2^*^ cells stably transfected with a metallothionein promoter plasmid expressing T7-tagged *rudra*, with or without treatment with CuSO_4_ and PGN, as indicated. IMD cleavage was analyzed by IP-IB (upper panel), while Relish phosphorylation and cleavage were analyzed by immunoblotting (in the middle two panels). The asterisk marks heavy chain detected by the secondary antibody. The lowest panel confirms Rudra expression with anti-T7 IB. (B) Northern blot of *Diptericin* and *rp49* expression levels in S2^*^ cells stably transfected with metallothionein promoter–driven transgenes expressing *PGRP-LCx*, *PGRP-LE*, or *imd*, with or without concurrent expression of *rudra*. Cells were treated with CuSO_4_ (+) or left untreated (−), and RNA was extracted after 6 hours. (C) Immunoblot analysis of Relish cleavage from S2^*^ cells stably transfected with metallothionein promoter expression plasmid for *Dredd*, with or without concurrent expression of FLAG-tagged *rudra*. CuSO4 was added, for 5 hours, to induce transgene expression, as indicated. Data are representative of at least three independent assays.

We next sought to determine the molecular mechanism(s) used by Rudra to control signal transduction. Relish, the NF-κB precursor protein essential for IMD triggered gene expression, is regulated by immune-induced cleavage and phosphorylation ([41],[42], unpublished data D.E-H. and N.S). Rudra expression prevented both the cleavage and phosphorylation of Relish (Figure 5A). Recently, we also discovered that *imd* protein is rapidly cleaved following immune stimulation (unpublished data, N.P. and N.S) and expression of *rudra* potently inhibits this cleavage (Figure 5A). These results suggest that Rudra functions upstream of Relish activation and IMD cleavage.

AMP gene expression can be triggered by ectopically expressing certain components of the IMD pathway. In particular, over-expression of either of the receptors, *PGRP-LC* or *PGRP-LE*, or *imd* is sufficient to drive AMP gene expression. Likewise, over-expression of the caspase *Dredd* is sufficient to drive Relish cleavage. To further analyze the position that Rudra acts in the IMD pathway, it was over-expressed with these signaling components in doubly selected stable cell lines. Rudra potently inhibited signaling induced by over-expression of the receptors PGRP-LC or PGRP-LE, but had no effect on the induction of *Diptericin* expression caused by IMD over-expression (Figure 5B). Likewise, Rudra did not inhibit Relish cleavage caused by over-expressing the caspase Dredd (Figure 5C). These results suggest that Rudra functions upstream of Dredd and IMD, but downstream of the receptors, and is consistent with binding data demonstrating an association between Rudra and either PGRP-LC or PGRP-LE.

**Figure 6:**
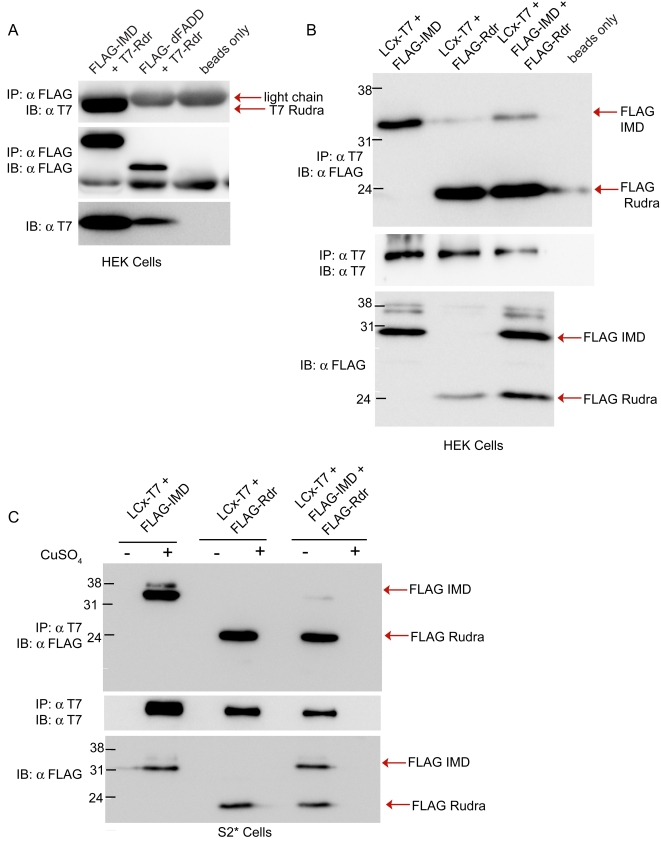
Rudra disrupts the interaction between PGRP-LCx and IMD. (A) IP-IB analysis of lysates of HEK cells transiently transfected with expression plasmids for FLAG-tagged *imd* or FLAG-tagged *dFADD* and T7-tagged *rudra*. Rudra interacted with IMD but not dFADD. (B, C) Similar co-immunoprecipitation experiments from lysates of HEK cells (B) or S2* cells (C) simultaneously co-transfected with T7 tagged *PGRP-LCx*, FLAG tagged *IMD* and/or FLAG-tagged *rudra*. Rudra interfered with the association between PGRP-LC and IMD. Data are representative of at least three independent assays. Data are representative of 3 independent experiments.

In addition to interacting with the receptors, Rudra avidly bound to IMD. The IMD association was detected by transient transfection/co-immunoprecipitation assays, in either S2* cells (data now shown) or HEK cells (Figure 6A). On the other hand, Rudra did not associate with dFADD, another factor known to interact with IMD. In all, these data argue that Rudra directly interacts with both IMD and the receptors PGRP-LC and PGRP-LE.

These results suggest two possible models for the inhibition of IMD signaling by Rudra: (1) Rudra may associate with both the receptor and its signaling adaptor (IMD), holding them together in an inactive confirmation; or (2) Rudra may interact with both PGRP-LC and IMD separately, disrupting the association between the receptor and its adaptor. To probe these possibilities, co-immunoprecipitation experiments were performed with lysates from cells co-transfected with *PGRP-LC* (T7 tag), *imd* (FLAG tagged) and/or *rudra* (also FLAG tagged). In assays with just the receptor and either IMD or Rudra, PGRP-LC interacted with either the adaptor or the inhibitor, in both *Drosophila* and human cells (Figure 6B, C). However, when all three proteins were simultaneously co-expressed, PGRP-LC and Rudra still robustly co-precipitated, but the association between IMD and the receptor was markedly reduced. These data suggest that Rudra interferes with the interaction between PGRP-LC and IMD, and this disruption provides a molecular mechanism explaining how Rudra down-modulates IMD signaling at the level of the receptor, consistent with the functional and binding data presented.

## Discussion

Recent work has shown that the intensity and duration of the immune response is tightly regulated in *Drosophila* [23],[24],[25],[27],[28]. Over-exuberant immune responses can be dangerous and the proper down modulation of immunity is important for health and fecundity [20],[22]. To keep the immune response properly modulated, the Toll and IMD pathways are controlled at multiple levels by multiple negative regulators. In this study, we have characterized a new negative feedback regulator of the IMD pathway. *rudra* transcript is rapidly induced following septic infection, and *rudra* mutant flies or *rudra* knockdown cells over-express antimicrobial peptides. In the case of *Erwinia carotovora carotovora* infection, this elevated level of AMP production leads to increased survival. A similar phenotype was reported for mutants lacking Caspar, which is thought to inhibit downstream signaling events [27]. The results presented here, in cells and flies, demonstrate that *rudra* is a key component in a negative feedback loop that keeps the IMD pathway in check.

In addition to these loss-of-function results, over-expression of *rudra* potently blocked signaling through the IMD pathway, both in cells and in flies. Moreover, we exploited this activity to analyze which steps in the IMD pathway are inhibited by Rudra. Using various molecular assays to monitor different PGN-induced events in the IMD pathway, we found that Rudra interfered with cleavage of IMD. Signaling mediated by receptor over-expression was also inhibited by Rudra, but this was not the case for signaling induced by over-expression of downstream components. Together, these data strongly support the notion that Rudra interferes with receptor function and is consistent with the association between Rudra and the receptors PGRP-LC or PGRP-LE.

Using assays in yeast, *Drosophila*, human cells and *in vitro*, Rudra was shown to interact directly with PGRP-LC and PGRP-LE. The interaction between PGRP-LE and Rudra required the RHIM-like domain of PGRP-LE, which is also critical for signaling by this receptor. However, the region through which PGRP-LC interacts with Rudra is less clear and likely involves multiple, partly redundant interfaces. Rudra also interacted with the *imd* protein. Moreover, Rudra interfered with the interaction between the receptor PGRP-LC and IMD, destabilizing the receptor signaling complex. From these results, we propose that Rudra is a negative feedback regulator that down modulates the IMD pathway by binding the receptors and interrupting the associations with their cognate signaling adaptor IMD. This regulatory loop is critical to properly regulate the immune response.

In agreement with the data presented here, Kleino et al. (2008) recently reported that *rudra*/CG15678 is a negative regulator of the IMD pathway, although they refer to this gene as *poor Imd response upon knock-in* (*pirk*). They showed that *rudra/pirk* is rapidly induced following infection, similar to the data presented here, and further demonstrated that *rudra* induction is dependent on Relish, both in cells and in flies. Using reporter assays in S2 cells, they found that Pirk inhibits IMD signaling but not the Toll pathway. With transgenic RNAi fly lines, they also found that knockdown of *pirk* caused the hyper-expression of the antimicrobial peptide genes. Also, flies over-expressing Pirk blocked the activation of the IMD pathway and were more susceptible infection. These results are consistent with the data presented here, although we have characterized a mutant allele of *rudra* and additionally show that this mutant exhibits enhanced protection against *Erwinia* infection. The data presented here also expand on the findings of Kleino et al. (2008) by showing that Rudra not only interacts with both PGRP-LC and IMD, but also that these interactions with Rudra disrupt the direct association between PGRP-LC and IMD. Kleino et al. (2008) reported that central portion of Rudra consists of two repetitive amino acid elements of unknown function and structure, which they named the Pirk domain. The Pirk domain is required for the interaction with IMD, but not with PGRP-LC. Rudra does not contain obvious homology to any other protein motifs, and no mammalian homologs are readily detected. [36].

Recently, multiple mechanisms involved in regulating the *Drosophila* immune response have come to light. Given that it is well-established that immune activation in flies has a cost, such as reduced fecundity [20],[22] and hypersensitivity to infection [23],[24],[27],[43],[44], it is not surprising that multiple negative regulatory circuits control the immune response. Similarly, in mammals, innate and adaptive immune responses are held in check by multiple mechanisms, in order to prevent inflammatory and autoimmune diseases while at the same time allowing an effective response to infection. Future studies will address the possible negative consequences of the lack of proper IMD regulation observed in the *rudra* mutant animals.

## Materials and Methods

### Reagents

Insoluble PGN from *E. coli* was purchased from Invivogen.

### Fly stocks and survival experiment

*rudra* mutant line, EY00723, was originally isolated by the *Drosophila* Genome Project gene disruption consortium and provided by the Bloomington Drosophila Stock Center. The flies were backcrossed for six generations to a *w^1118^* strain in order to isogenize. In all experiments, *rudra^EY00723^* mutants were compared to isogenic *w^1118^* animals. *PGRP-LE^112^;;PGRP-LC* ^Δ*E*^, double mutant flies were reported previously [45]. Survival experiments were performed with 60 flies at 29°C, following infection by pricking in the abdomen with a microsurgery needle dipped into a concentrated pellet *of Erwinia carotovora carotovora 15* [24]. Surviving flies were transferred to fresh vials and counted daily, until all wild-type flies died. Kaplan-Meier plots are presented and P-values were calculated by log-rank test using GraphPad Sigma Plot.

### RNA analysis and RT-PCR

Total RNA from flies or cultured cells was isolated with the TRIzol reagent (Invitrogen) as described previously [33]. Expression of *Diptericin*, *Attacin*, *Cecropin* and the control *rp49* (ribosomal protein) was analyzed by Northern blotting [33]. Northern blots were quantified with a phosphoimager (Fuji) and AMP gene expression was normalized to *rp49* levels. For qRT-PCR, RNA was DNase treated and re-extracted with phenol-chloroform. cDNA was synthesized using Superscript II (Invitrogen) and quantitative PCR analysis was performed on a DNA engine Opticon 2 cycler (MJ Research, Watertown MA) using SYBR Green (Biorad). The specificity of amplification was assessed for each sample by melting curve analysis and relative quantification was performed using a standard curve with dilutions of a standard. The quantified data was normalized to *rp49* levels. In all S2*-based cell experiments, cells were pre-treated with 1 mM 20-hydroxyecdysone for 24 to 40 hr before treatment with 500 mM CuSO4 and/ or PGN (100 ng/ml).

### RNAi experiments

dsRNA was generated and purified as reported previously [46]. Cells were split 24 hours after transfection to 1.0×10^6^/mL and then were treated with 1 mM 20-hydroxyecdysone. After 24 hours, cells were treated (or left untreated) with PGN (100 ng/ml) for various time, as indicated.

### Co-immunoprecipitation and immunoblotting assays

*In vitro* translation was performed following the protocol of the manufacturer (Promega). Immunoprecipitations were carried out with rabbit anti-T7 (Bethyl labs) in lysis buffer (20 mM Tris at pH 7.6, 150 mM NaCl, 2 mM EDTA, 10% Glycerol, 1% Triton X-100, 1 mM DTT, NaVO4, glycerol 2-phosphate and protease inhibitors). For immunoprecipitation from cells, Schneider S2* cells were first transfected by calcium phosphate method with appropriate expression plasmids. Cells were split 24 hours after transfection to 1.0×10^6^/mL and 24 hours later, were treated with 500 µM copper sulphate for 5 hr, when necessary, for expression from the metallothionein promoter. Immunoprecipitations were performed in lysis buffer and analyzed by SDS-PAGE followed by immunoblot analysis with anti-T7 MAb (Novagen), anti-V5 (Sigma), anti-IMD (gift of J.-M. Reichhardt) or anti-Flag (Sigma) antibodies. Stable cell lines and immunoblotting were performed as described previously [33]. The generation and characterization of phospho-specific Relish antibody will be detailed elsewhere (D. E.-H. and N.S., unpublished data).

### Transgenesis and analysis of UAS-*rudra* and genomic rescue strains

For the UAS transgenic, the *rudra* ORF was amplified by PCR and subcloned into the *Eco*RI and *Bgl*II sites of pUAST. For genomic rescue, a BAC clone (Drosophila Resource Center [47]) was used as a template to amplify a 4.5 Kbp genomic fragment containing the complete *rudra* locus plus flanking sequences, which was then cloned into the *Eco*RI and *Bam*HI sites of pCaSpeR [48]. After sequence verification, standard techniques were used for P-element–mediated transformation at the MGH Drosophila transgenics facility. For immune stimulation assays, adults (males and females in equal numbers), were infected by pricking in the abdomen with a microsurgery needle dipped into a concentrated pellet of *E. coli (1106)*, RNA was extracted 8 h later, and assayed by Northern blotting.

### Stable cell lines

The *rudra* gene was cloned into pRmHa3 vector by standard methods to create constructs expressed from the metallothionein promoter. The constructs were then transfected into S2* cells in conjunction with pHs-Neo at a ratio of 50∶1; stable transfectants were then selected with G418 (1 mg/ml). For double stable cell lines, the *rudra* expression plasmid was transfected into S2* cell lines that were previously selected to carry plasmids expressing either *PGRP-LC*, *PGRP-LE*, *IMD* or *Dredd*. The *rudra* plasmid was selected with a second selectable marker, either G418 (1 mg/ml) or hygromycin (20 U/ml), as appropriate.

## Supporting Information

Figure S1 PGRP-LE and Rudra interact *in vitro* Co-immunoprecipitation of in vitro co-translated PGRP-LE and Rudra. Co-immunoprecipitation was performed using anti-FLAG antibodies with ^35^S-methionine labeled *in vitro* translated T7-Rudra and FLAG-PGRP-LE.
(3.77 MB TIF)

Figure S2 Rudra interacts with all the deletion mutants spanning the cytoplasmic domain of PGRP-LCx. IP-IB analysis of lysates from S2* cells transiently transfected with metallothionein promoter expression plasmids encoding T7-tagged *PGRP-LCx* (wild-type and deletion mutants) and FLAG-tagged *rudra* with or without CuSO4 treatment, as indicated. Lower diagram indicates the regions deleted in each mutant form of PGRP-LC.
(0.87 MB TIF)

Figure S3 Rudra inhibits IMD signaling but not the Toll pathway. Northern blot of *Drosomycin* and *Diptericin* expression in S2* cells stimulated with SPZ-C106 or PGN, respectively, with *rp49* as a loading control. Cells expressing *rudra*, from the actin promoter, failed to respond to PGN but displayed robust SPZ-induced *Drosomycin* expression. Stimulation time as indicated.
(4.55 MB TIF)
