# Supplementary figures and images for: Rudra Interrupts Receptor Signaling Complexes to Negatively Regulate the IMD Pathway
Source: PLoS Pathog. 2008 Aug 8;4(8):e1000120. doi: 10.1371/journal.ppat.1000120 (PMC2483946; doi:10.1371/journal.ppat.1000120)

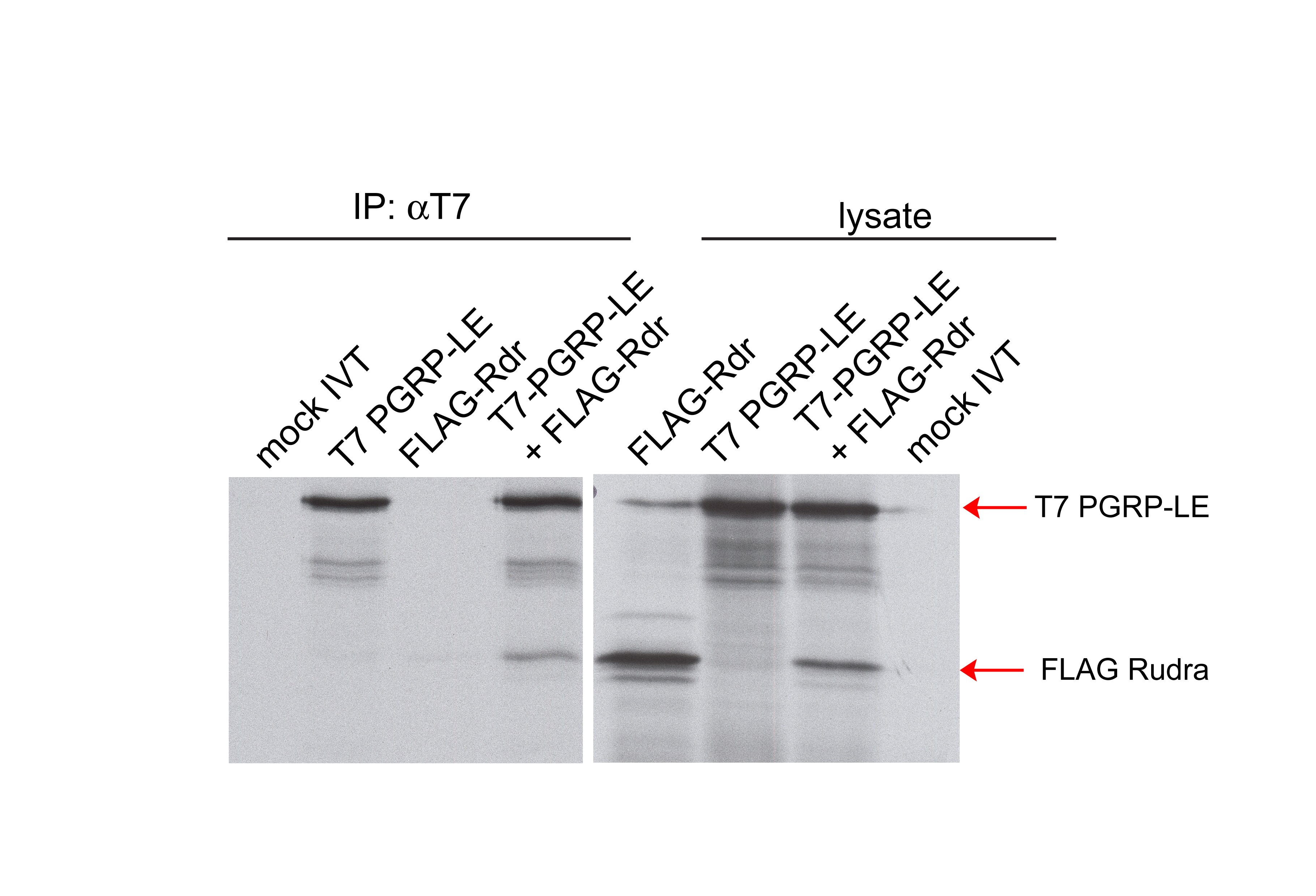

Supplement: Figure S1 — PGRP-LE and Rudra interact in vitro Co-immunoprecipitation of in vitro co-translated PGRP-LE and Rudra. Co-immunoprecipitation was performed using anti-FLAG antibodies with 35S-methionine labeled in vitro translated T7-Rudra and FLAG-PGRP-LE. (3.77 MB TIF) [file ppat.1000120.s001.tif]

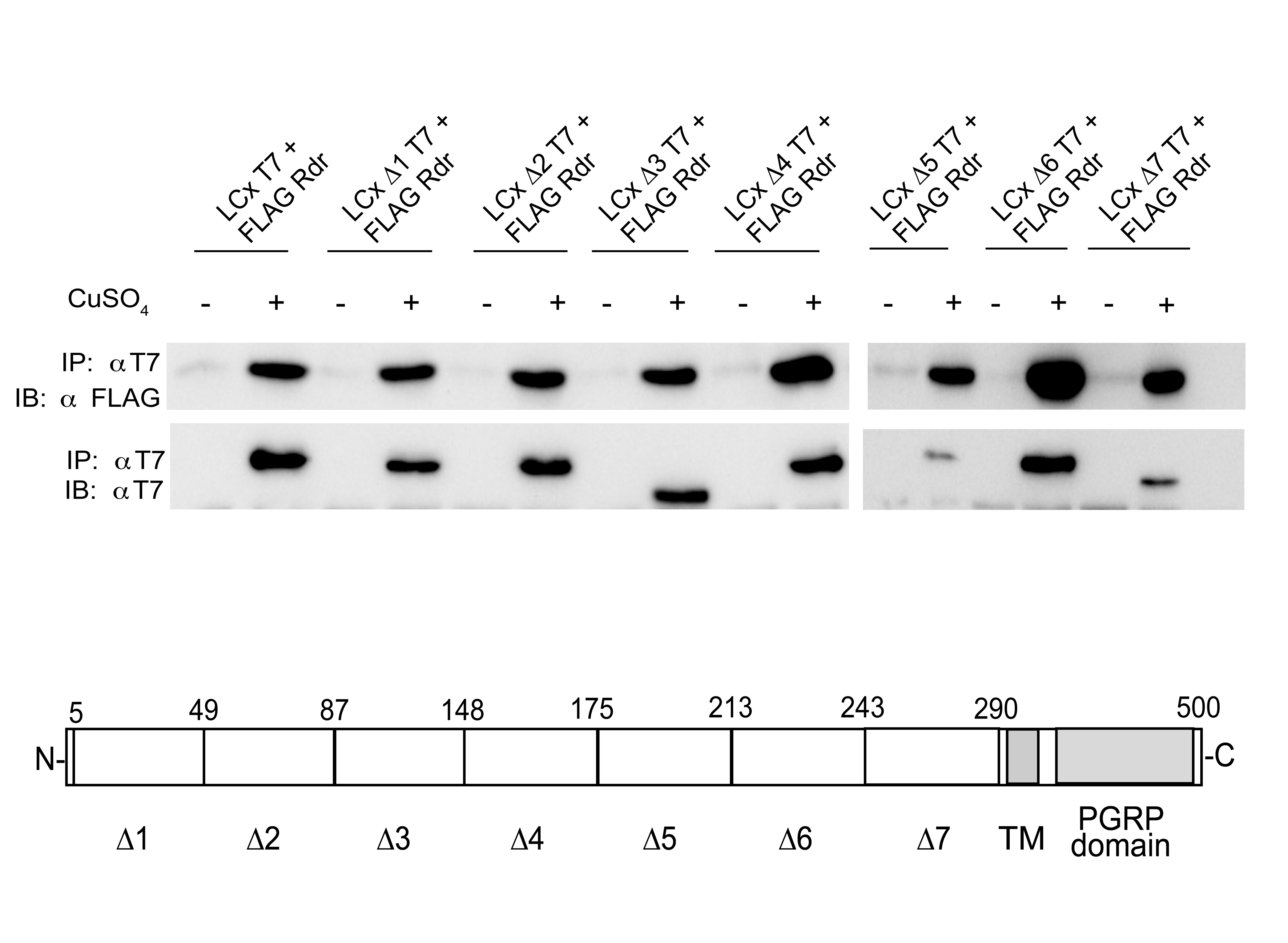

Supplement: Figure S2 — Rudra interacts with all the deletion mutants spanning the cytoplasmic domain of PGRP-LCx. IP-IB analysis of lysates from S2* cells transiently transfected with metallothionein promoter expression plasmids encoding T7-tagged PGRP-LCx (wild-type and deletion mutants) and FLAG-tagged rudra with or without CuSO4 treatment, as indicated. Lower diagram indicates the regions deleted in each mutant form of PGRP-LC. (0.87 MB TIF) [file ppat.1000120.s002.tif]

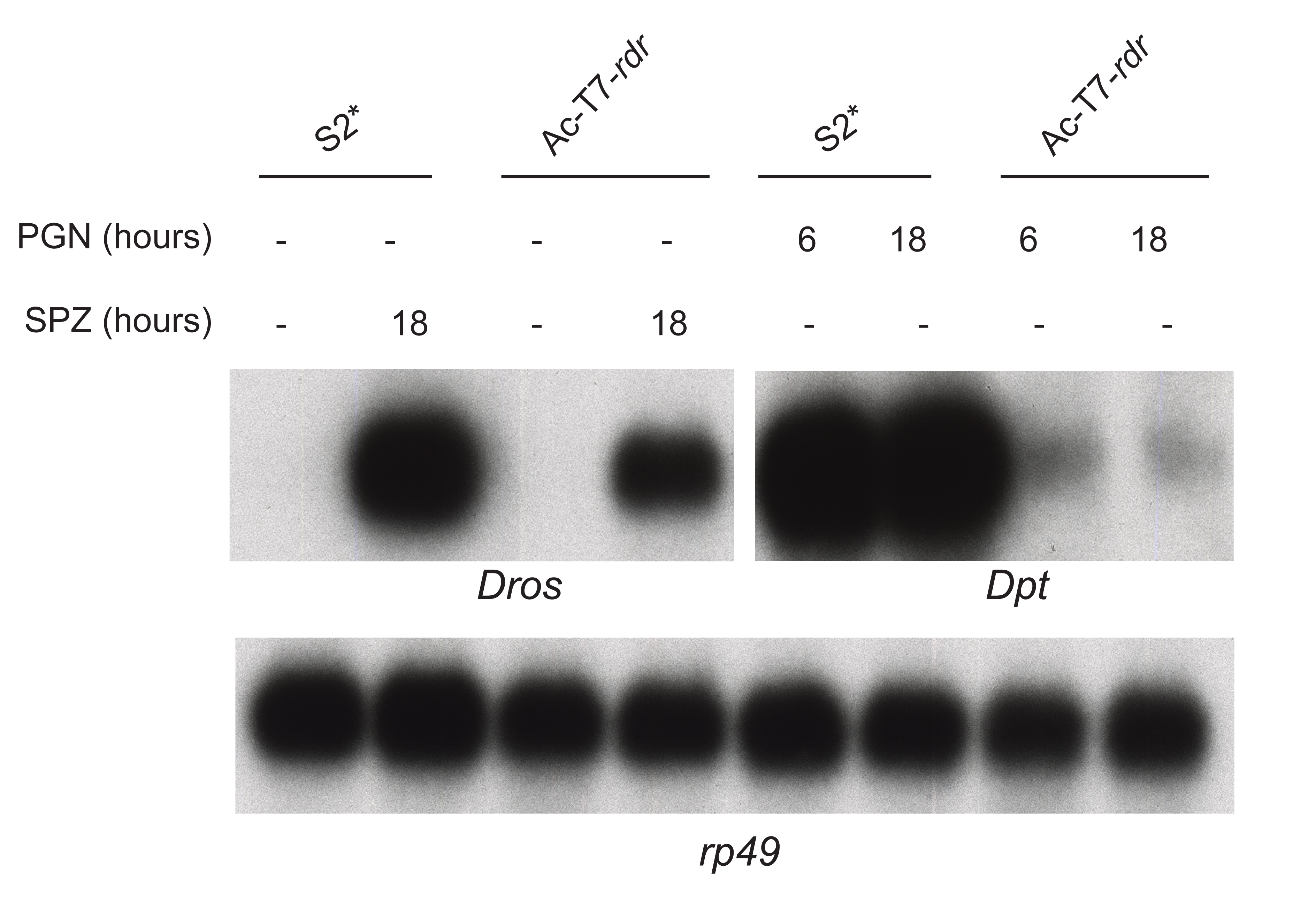

Supplement: Figure S3 — Rudra inhibits IMD signaling but not the Toll pathway. Northern blot of Drosomycin and Diptericin expression in S2* cells stimulated with SPZ-C106 or PGN, respectively, with rp49 as a loading control. Cells expressing rudra, from the actin promoter, failed to respond to PGN but displayed robust SPZ-induced Drosomycin expression. Stimulation time as indicated. (4.55 MB TIF) [file ppat.1000120.s003.tif]
